# Audio Feedback Associated With Body Movement Enhances Audio and Somatosensory Spatial Representation

**DOI:** 10.3389/fnint.2018.00037

**Published:** 2018-09-04

**Authors:** Anna Vera Cuppone, Giulia Cappagli, Monica Gori

**Affiliations:** Unit for Visually Impaired People (U-VIP), Istituto Italiano di Tecnologia, Genoa, Italy

**Keywords:** feedback, perception, proprioception, audio, sensorimotor

## Abstract

In the last years, the positive impact of sensorimotor rehabilitation training on spatial abilities has been taken into account, e.g., providing evidence that combined multimodal compared to unimodal feedback improves responsiveness to spatial stimuli. To date, it still remains unclear to which extent spatial learning is influenced by training conditions. Here we investigated the effects of active and passive audio-motor training on spatial perception in the auditory and proprioceptive domains on 36 healthy young adults. First, to investigate the role of voluntary movements on spatial perception, we compared the effects of active vs. passive multimodal training on auditory and proprioceptive spatial localization. Second, to investigate the effectiveness of unimodal training conditions on spatial perception, we compared the impact of only proprioceptive or only auditory sensory feedback on spatial localization. Finally, to understand whether the positive effects of multimodal and unimodal trainings generalize to the untrained part, both dominant and non-dominant arms were tested. Results indicate that passive multimodal training (guided movement) is more beneficial than active multimodal training (active exploration) and only in passive condition the improvement is generalized also on the untrained hand. Moreover, we found that combined audio-motor training provides the strongest benefit because it significantly affects both auditory and somatosensory localization, while the effect of a single feedback modality is limited to a single domain, indicating a cross-modal influence of the two domains. Therefore, the use of multimodal feedback is more efficient in improving spatial perception. These results indicate that combined sensorimotor signals are effective in recalibrating auditory and proprioceptive spatial perception and that the beneficial effect is mainly due to the combination of auditory and proprioceptive spatial cues.

## Introduction

For humans, the ability to spatially localize stimuli in space is fundamental in daily life activities, when people interact with the environment by reaching, manipulating, or moving objects. Several studies indicate that among the sensory modalities, vision is fundamental in guiding the maturation of space representation in the brain mainly because it provides an immediate and complete representation of the environment in a single frame (Tinti et al., 2006). As a consequence, vision typically dominates spatial perception by providing the most accurate and reliable information about the spatial properties of the external world (Alais and Burr, 2004; Gori et al., 2012a). Indeed, the lack of visual experience in the first period of life impacts on the acquisition of spatial abilities and visually impaired individuals typically show compromised auditory (Zwiers et al., 2001; Lewald, 2002; Eimer, 2004; Kolarik et al., 2013; Gori et al., 2014), proprioceptive and motor skills (Pasqualotto and Newell, 2007; Postma et al., 2008; Cappagli and Gori, 2016).

Rehabilitation training aiming at restoring spatial abilities in the case of disabilities is typically designed to stimulate one function at a time, e.g., auditory or tactile functions in the case of blindness and the proprioceptive function in the case of motor diseases. For instance, in the case of blindness, sensory substitution technologies exploit the intact senses (audition or touch) separately, to provide spatial information and improve spatial representations. In particular, sensory substitution devices translate visual information into auditory or tactile stimuli that visually impaired individuals learn to process across long extensive training (Auvray and Myin, 2009; Velázquez, 2010; Proulx et al., 2014). Tactile and auditory sensory substitution devices respectively providing electrical/vibratory stimulation applied to the skin of a part of the body (e.g., abdomen, tongue, etc.) or auditory stimulation through changes in pitch and amplitude, can improve simple form recognition (Arno et al., 1999, 2001; Cronly-Dillon et al., 1999, 2000; Kaczmarek and Haase, 2003; Pollok et al., 2005) and localization abilities (Renier et al., 2005; Jansson, 2009). Nonetheless, since all the substitution devices developed so far rely on unisensory (visual-to-auditory or visual-to-tactile) stimulation, it might be that sensory training based on bimodal stimulation lead to stronger long-lasting reinforcement of residual perceptual abilities.

In a similar way, sensory substitution or augmentation is used to enhance sensorimotor function of subjects with motor deficits. Indeed, balance training with a vibrotactile neurofeedback system improved the overall stability in patients with Parkinson’s disease, reducing the number of falls and improving the body sway (Rossi-Izquierdo et al., 2013). In case of neurological diseases such as stroke and spinal cord injury, several categories of strategies for robotic therapy devices with haptic and visual feedback show a significant reduction of motor impairment, assessed with standard clinical outcome measures (Marchal-Crespo and Reinkensmeyer, 2009). Moreover, it has been shown that augmented feedback benefits sensorimotor learning on healthy subjects, highlighting a superior effect of multimodal feedback (Sigrist et al., 2013). Nonetheless, in all these studies the non-proprioceptive feedback (auditory or visual stimuli) is barely informative respect to spatial coordinates because it is not spatially congruent with the proprioceptive feedback; thus, spatial information was provided only through proprioception.

In addition, only few studies assessed the influence of multimodal training on sensorimotor function, in particular on proprioception (the ability to perceive body position) which is fundamental for motor control (Proske and Gandevia, 2012). In this respect, it is shown that a sensorimotor training with additional vibrotactile feedback about movement errors improves spatial acuity of the wrist joint position sense, indicating that such training affects not only motor functions, but also proprioceptive domain (Cuppone et al., 2016). Moreover, several reports indicate that error signals given as kinesthetic cues can cause visuomotor perturbation to which individuals gradually adapt, indicating that proprioception can contribute to the mechanisms underlying sensory adaptation (Sarlegna et al., 2010). Similarly, when auditory feedback is provided as an error signal, it reduces endpoint error in healthy subjects by 17%–31% (Hocherman et al., 1988; Hocherman, 1993), improving the sense of body position. Moreover, it has been shown that real-time auditory feedback in the form of timbre variations (Thoret et al., 2016) or sonification (Danna et al., 2015) can substantially alter motor performance.

Finally, the training condition (e.g., active vs. passive movement) influences sensorimotor learning. It has been shown that active proprioceptive training coupled visual feedback reduces proprioceptive errors immediately after training, suggesting that rehabilitation interventions including active movements component are superior to interventions that only employ passive limb motion (Beets et al., 2012). However, as hypothesized by the authors, the superiority of active over passive training in the presence of augmented feedback can be due to the involvement of the visual feedback in active processes of error detection/correction and planning. Therefore, to confirm this superiority, the condition without visual feedback in both modalities should be considered.

According to such results it can be claimed that perceptual training enhancing auditory and proprioceptive functions are useful for the development of spatial cognition, but reports on the efficacy of such training interventions and modalities vary widely. We recently developed a new device called Audio Bracelet for Blind Interaction (ABBI; Finocchietti et al., 2015; Gori et al., 2016) which is an audio bracelet that provides sensory feedback of body movements by emitting sounds at movement onset. We recently showed that the use of the device for 3 months in blind children from 3 years to 5 years (Cappagli et al., 2017b) and for only 2 min in adults (Finocchietti et al., 2017) improves their spatial representation. These results suggest that sensory-motor training based on the strengthening of auditory and tactile functions with motor command can lead to tangible enhancements of perceptual functions that have an immediate impact on daily functioning (Gori et al., 2016). To date it still remains unclear whether: (i) active and passive multimodal (audio-proprioceptive) training conditions provide the same level of spatial enhancement (meant as the improvement in stimuli localization) in the auditory and proprioceptive domain; (ii) spatial enhancement deriving from unimodal (audio or proprioceptive) training conditions is visible only in the specific domain targeted by the training or extend also to the untrained domain; and (iii) spatial enhancement deriving from unimodal and multimodal training conditions generalize to the untrained side of the body.

In this study, we investigated the influence of active and passive audio-motor training on audio and proprioceptive space representations. To this end, we compared the influence on spatial accuracy of an active condition in which subjects voluntary explored the workspace and a passive condition in which subject’s movements were guided by the experimenter. We expect that active training condition results in better spatial enhancement due to the fact that motor command modulated tactile and proprioceptive perception (Sciutti et al., 2010; Gori et al., 2012b; Tomassini et al., 2014). Moreover, we hypothesize that multimodal training with audio and proprioceptive feedback can result in improved spatial perception both for external (i.e., audition) and body (i.e., proprioception) stimuli. In order to understand whether positive effects of the training are generalized on the untrained part, we assessed spatial perception after the training both on the trained and untrained arms. We expect that spatial enhancement resulting from unimodal training is specific for the targeted domain, that is unimodal auditory training improves auditory spatial perception and unimodal proprioceptive training improves proprioceptive spatial perception.

## Materials and Methods

### Participants

**Figure 1:**
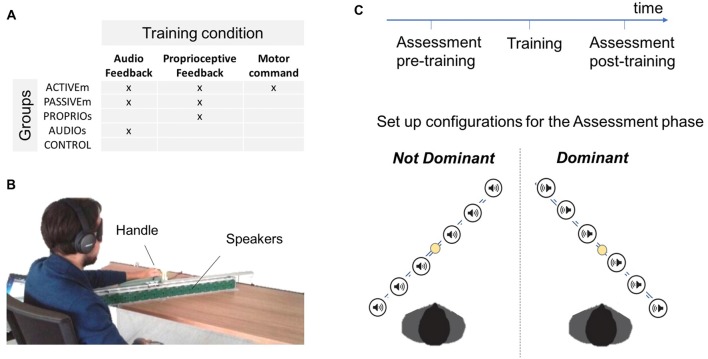
Experimental protocol. **(A)** Description of different training groups depending on the training condition: active or passive movements with independent or combined audio and proprioceptive feedback. **(B)** Experimental set up. **(C)** Description of the experimental protocol and schema of the two configurations used in the Assessment test.

The study involved 40 subjects (age: 29.08 ± 5.71) with no known neuromuscular disorders and naïve to the task. The participants were divided in four groups: the Active multimodal feedback group (**ACTIVEm**, *n* = 8) which performed active movements by receiving the audio and proprioceptive feedback; the passive multimodal feedback group (**PASSIVEm**, *n* = 7) which was passively guided during training by receiving the audio and proprioceptive feedback; the only proprioceptive feedback group (**PROPRIOu**, *n* = 9) and the only audio feedback group (**AUDIOu**, *n* = 9) who performed training in a passive condition by using a single feedback information, the proprioceptive and the audio feedback respectively (unimodal conditions); the control group (**CONTROL**, *n* = 7) who did not perform any training. The training conditions are summarized in Figure 1A, while the details are provided as follow.

### Experimental Protocol

The study presented a pre-post test intervention (*Assessment phase*) where two different aspects of spatial cognition were evaluated. The first task assessed subject’s ability to localize and reach sounds in space (*Reaching of auditory cue task*) while the second task tested the ability to reproduce a position in space (*Position matching task*). In both tasks participants were blindfolded. Each subject performed the assessment tests with both arms (*Dominant* and *Not Dominant*) but only the dominant arm was trained during the *Training phase*. The pre-post tests were performed in a random order. An overview of the design is shown in Figure 1C and procedure details are described below.

#### Experimental Set Up

The set up showed in Figure 1B utilized a set of 16 loudspeakers (dimension of each speaker 53 mm × 53 mm) placed in a metallic support. Each speaker surface was covered by 5 × 5 tactile sensors (1 cm × 1 cm each) that can register the position of the contact and provide accurate information about spatial errors (measure accuracy = 0.5 cm). The system was controlled by a workstation and the software environment was implemented on Matlab. The serial communication between the workstation and the loudspeakers was bidirectional and it allowed the selected loudspeaker to execute the sonorous stimulus and to register the position of the touched sensor.

##### Pre-post Test Proprioceptive and Audio Localization Assessment

###### Reaching of Auditory Cue Task

In order to test the ability of spatial localization in the auditory domain, we asked participants to reach a sonorous stimulus provided by one of the six selected speakers (Figure 1B). The sonorous stimulus was a pink noise with a duration of 1 s. The set of 16 loudspeakers was placed on the desk in front of the subject along a line inclined with an angle of 45° respect to the frontal axis of the human body. The center of the set of loudspeaker was 20 cm far from the center of the body in order to allow subjects to easily reach far positions. The subject hold a handle able to slide on a metallic rail positioned on the set of loudspeakers. After the end of the stimulus, the participant moved his arm in order to reach the source of the auditory cue. When the subject confirmed the position, the experimenter touched the corresponding point on the loudspeaker behind the subject’s position. We asked participants to be accurate in auditory localization without giving a time constraint.

The six target positions were equally distributed along the direction of movement in order to test the entire workspace and corresponded to the loudspeakers 3, 5, 7, 10, 12, 14 (the first speaker was the closest to the subject in each configuration). Each target was presented five times, for a total of 30 trials. The target sequence presentation was randomized.

###### Position Matching Task

In order to test proprioceptive function, subjects performed an ipsilateral Position matching task (Goble, 2010) in both configuration (Dominant and Not Dominant). The experimenter moved the subject’s arm from the starting position (loudspeaker 1) to the desired position and after 1 s moved it back to the initial position. The subject had to replicate the previous experienced position and once he confirmed the end of reaching movement, the experimenter touched the loudspeaker surface on the correspondent point in order to register the matching position. The targets location was identical to the previous task, as the number of repetition and the total number of trials (*n* repetitions = 5; *n* trial = 30); the target sequence was randomized. We asked participants to be accurate in reaching the position without giving a time constraint.

##### Training

Subjects were divided in different groups depending on the training condition (Figure 1A). For each training condition, subjects were blindfolded in order to eliminate the contribution of visual feedback and were trained for 10 min divided in four blocks of 2.5 min each. The participants were trained on their dominant hand. The active training performed by the ACTIVEm group consisted of a free exploration of the workspace. The subject grasped a handle which slid on the rail (Figure 1B) and simultaneously received an audio feedback of a pink noise provided by the ABBI device (Finocchietti et al., 2015; Gori et al., 2016). It has been created to provide an audio feedback about body movements to help visually impaired people, more specifically children, to build a sense of space. The ABBI was programed in remote control therefore the audio command was triggered by the experimenter using a mobile phone. In the passive condition (PASSIVEm and PROPRIOu), the participants’ arm was passively moved by the experimenter in two ways: (a) continuous movement; (b) discrete small movements of 5 cm with 1 s of stop. The PASSIVEm group performed this training by receiving the additional auditory feedback (ABBI was positioned on the subject’s wrist), while the PROPRIOu relied only on the proprioceptive information. On the contrary, subjects of the AUDIOu group did not move their arm but received the auditory feedback provided by the ABBI device that was moved by the experimenter in the same modality used for passive condition.

#### Measurements and Statistical Analysis

In order to evaluate the accuracy of reaching a sonorous stimulus and matching a position in the space we analyzed the distance error (in millimeters) between the subject position and the target, and we extracted the Matching Error (ME) variable.

ME represents a measure of trueness or its inverse, bias. It is defined as the Euclidean distance between the target and the final arm position.

(1) $$ME=\sum_{i=1}^{N}{\left(x_{EE}-x_{TG}\right)}^{2}$$

where, *N* are the number of Target repetitions (5), *x*_EE_ is the subject’s final position and *x*_TG_ is the Target position. These variable values were then averaged across targets.

To analyze the effect of training on audio and proprioceptive localization, we evaluated the Normalized change of ME as follow:

(2) $$Normalized\;change\;of\;ME=nME=\frac{ME_{Pre}-ME_{Post}}{ME_{Pre}}100$$

We applied an analysis of variance (ANOVA) between groups before training in order to evaluate differences between samples. Consequently, we evaluated the effect of different training conditions by considering the Normalized change of ME and performing an ANOVA between groups. To evaluate if the change with training was significant within group, we then applied the *t*-test.

## Results

We investigated the global effect of different training modalities (five subject’s groups) in each domain (Auditory and Proprioceptive) for the trained and untrained hand. The analysis has revealed a not significant difference among groups for the ME in the pre-training evaluation (ANOVA Group factor *p* > 0.05). We then performed a second analysis considering the performance variation (nME) among groups which revealed a significant difference among groups for the trained hand (Auditory Domain: ANOVA Group effect *p* = 0.0014, Proprioceptive Domain: ANOVA Group effect *p* = 0.0009). For this reason, we then performed a *post hoc* *t*-test to investigate which groups had a significant variation in performance between the pre and post test evaluation. However, this analysis was divided into two steps, with the intention to clarify the effect of: (a) active and passive multimodal conditions; and (b) unimodal and multimodal conditions effects. The consequent results are described in the following two sections.

### Effect of Active and Passive Training Conditions

**Figure 2:**
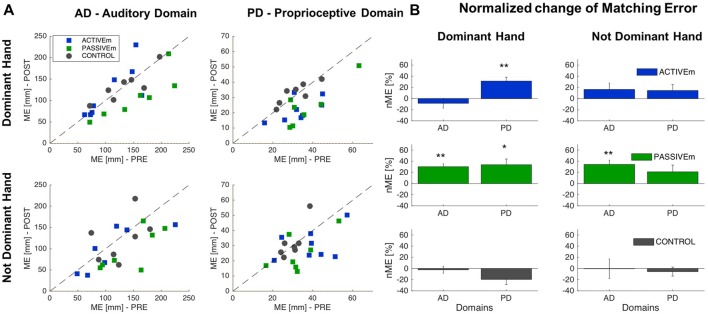
Comparison of Active and Passive training conditions. **(A)** Matching Error (ME) values of Auditory and Proprioceptive Domain related to the Dominant (trained side) and Not Dominant (untrained side) hand for the ACTIVEm, PASSIVEm and CONTROL group. Each dot represents ME value of single subject in the PRE training phase (*x* axis) and POST training phase (*y* axis). **(B)** Relative ME change (mean ± SE) for the ACTIVEm, PASSIVEm and CONTROL group for proprioceptive and auditory domain, evaluated for Dominant and Not Dominant hand. *p < 0.05, **p < 0.01.

We analyzed the effect of sensorimotor training with proprioceptive and auditory feedback on proprioceptive and auditory localization in two conditions: the active training, in which subjects voluntary explored the workspace and the passive condition in which subject’s arm was moved by the experimenter. We compared the ACTIVEm, PASSIVEm and CONTROL group’s performance. The results confirmed that the groups at the baseline (before training) presented the same accuracy (ANOVA: Group factor *p* > 0.05) in localization of sonorous stimuli (auditory domain) and matching a position (proprioceptive domain), for both hands (Dominant and Not Dominant); values are reported in Table 1 and for the *t*-test as *post hoc* *analysis* we applied the Bonferroni correction (0.05/3 = 0.017). Figure 2 reports the training results for ACTIVEm, PASSIVEm and CONTROL groups. When we consider the performance in the Auditory Domain for the trained side (Dominant hand), only PASSIVEm group decreased the ME and seven by eight subjects improved their accuracy in sound localization (Figure 2A). A similar behavior was reported for the untrained side, where only PASSIVEm group shows an error decrease. The relative improvement of accuracy evaluated by the nME for this group was indeed 30.2% ± 5.16% (*p* = 0.001) and for the untrained side was 34.17% ± 7.7% (*p* = 0.004). Both CONTROL and ACTIVEm group did not decrease their accuracy in reaching the sound source neither for trained nor untrained side (nME CONTROL *p* > 0.05). When we consider the training effect on the proprioceptive function, both ACTIVEm and PASSIVEm training conditions have a beneficial effect, but the improvement was limited on the trained side. Therefore, the nME for the Dominant hand was 31.35% ± 6.97% for ACTIVEm group (*p* = 0.003) and 33.62% ± 10.28% for PASSIVEm (*p* = 0.017). The improvement was not present for the Not Dominant hand (*p* > 0.05). The CONTROL group did not show any change in the ME with training (*p* > 0.05).

**Table 1:** Matching error (ME) values (mean ± SE) are reported for all subject’s groups: ACTIVEm, PASSIVEm, CONTROL PROPRIOu and AUDIOu before training (Assessment pre-training).

Groups	Auditory domain		Proprioceptive domain	
	Dominant hand [mm]	Not Dominant hand [mm]	Dominant hand [mm]	Not Dominant hand [mm]
ACTIVEm	108.7 ± 14.7	130.2 ± 26.9	32.9 ± 3.4	38.5 ± 4.3
PASSIVEm	154.3 ± 21.5	145.9 ± 17.3	34.7 ± 2.7	31.9 ± 3.4
CONTROL	134.1 ± 15.7	126.6 ± 14.3	2.8 ± 2.6	29.8 ± 1.9
PROPRIOu	156.2 ± 12.6	170.1 ± 31.8	30.9 ± 2.9	34 ± 1.6
AUDIOu	174.6 ± 18.9	144.70 ± 9.5	31.4 ± 1.3	31.7 ± 2.3

*The error values are related to the sonorous stimuli localization evaluated by the Reaching of Auditory Cue task and to the matching of a spatial position evaluated in the Position matching task. Values are reported for the Dominant (trained) space and the Not Dominant (untrained) space*.

### Effect of Combined and Independent Audio-Proprioceptive Feedback

In order to evaluate the effect of the audio and proprioceptive feedback, we compared the performance of multimodal feedback training (PASSIVEm) with the other two conditions where a single feedback was provided: the training with solely audio feedback without any motor and proprioceptive component (AUDIOu) and the training with only proprioceptive information (PROPRIOu). The results confirmed that the groups at the baseline (before training) presented the same accuracy (ANOVA: Group factor *p* > 0.05) in localization of sonorous stimuli (auditory domain) and matching a position (proprioceptive domain), for both hands (Dominant and Not Dominant hand); values of PASSIVEm, PROPRIOu and AUDIOu groups are reported in Table 1.

**Figure 3:**
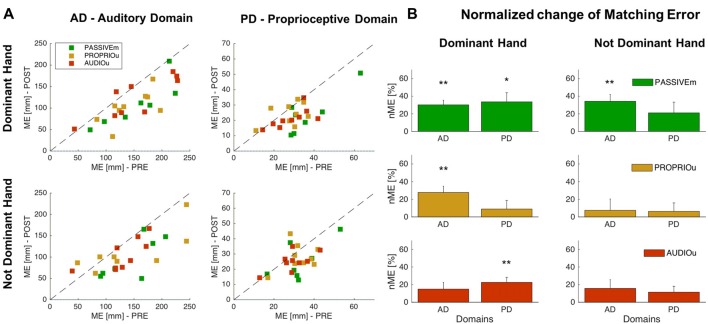
Comparison of combined and independent audio-motor feedback.** (A)** ME values of Auditory and Proprioceptive Domain related to the Dominant (trained side) and Not Dominant (untrained side) hand for the PASSIVEm, PROPRIOu (group with only proprioceptive feedback) and AUDIOu (group with only auditory feedback) group. Each dot represents ME value of single subject in the PRE training phase (*x* axis) and POST training phase (*y* axis). **(B)** Relative ME change (mean ± SE) for the PASSIVEm, and AUDIOu group for proprioceptive and auditory domain, evaluated for Dominant and Not Dominant hand. *p < 0.05, **p < 0.01.

We compare the effect of training with combined multimodal feedback (PASSIVEm) with single feedback (AUDIOu and PROPRIOu) by considering as significant level *α* = 0.05/3 = 0.017. We found that the first condition highly affects the proprioceptive and auditory domains. As mentioned before, the significant change of ME for PASSIVEm group occurred for both domains with the Dominant hand, and the learning was generalized to the Not Dominant side for the Auditory Domain (Figures 2, 3). On the other hand, training without audio feedback (PROPRIOu condition) was not enough to modify significantly proprioceptive function (Proprioceptive domain—nME: 8.9% ± 9.7% *p* = 0.38) and similarly the training with only audio feedback and without any movement (AUDIOu) tended to change the accuracy in sound source localization, but not significantly (Auditory domain—nME 14.9% ± 7.6% *p* = 0.08). A mutual effect in the trained side was found between the two domains: proprioceptive training influenced auditory perception (PROPRIOu group—Auditory Domain nME: 27.8% ± 7.1% *p* = 0.005) and vice versa audio training affected proprioceptive function (22.5% ± 5.6% *p* = 0.004). Results are shown in Figure 3 An overview of the results of the entire study is presented by Table 2.

**Table 2:** Summary of training conditions and training results for each group of subjects for Auditory and Proprioceptive domains and for Dominant and Not Dominant hands.

Groups			ACTIVEm	PASSIVEm	PROPRIOu	AUDIOu	CONTROL
Training condition	Dominant hand	Audio feedback	x	x	-	x	-
		Proprioceptive feedback	x	x	x	-	-
		Motor command	x	-	-	-	-
Training results	Dominant hand	Auditory domain	-	x	x	-	-
		Proprioceptive domain	x	x	-	x	-
	Not dominant hand	Auditory domain	-	x	-	-	-
		Proprioceptive domain	-	-	-	-	-

*In training results the significant changes due to training are indicated with an x, while for training condition x indicates the presence of a specific feedback*.

## Discussion

It is well known that we live in a multisensory world in which contingencies of sensory information are typically integrated to enhance perception. For this reason, it has been suggested that multimodal training protocols might be more effective than unimodal training protocols for learning purposes, since they better reflect natural settings and provide redundancy exposure that typically supports perceptual development (Shams and Seitz, 2008). Scientific reports indicating that early brain areas are particularly sensitive to multisensory interactions (Ghazanfar and Schroeder, 2006; Driver and Noesselt, 2008) seem to confirm this view. Understanding the impact of multimodal stimulation on perceptual abilities becomes of critical importance in the case of sensory and motor dysfunctions. For instance, in the case of blindness, there is the need to solicit intact senses, like audition and touch, with specific sensory training in order to recover from spatial impairments caused by the lack of vision (Gori et al., 2014, 2016). Similarly, in the case of motor dysfunctions, multimodal trainings focused on sensorimotor coupling might be employed to improve specific perceptual functions (Aman et al., 2014). Therefore, it is fundamental to understand the effect of multisensory training on spatial representation of our body and of the external environment.

The novelty of this study was to report the effect of multimodal sensorimotor training with additional external auditory feedback in different training conditions (active movements vs. passive guided movements) considering two sensory domains: audition and proprioception.

### Active vs. Passive Multimodal Training Conditions

The first main finding of this work is that both active and passive multimodal conditions are beneficial for proprioceptive function, but only the passive training enhances the ability to perceive the sound location even with the untrained arm. This might suggest that while the association of auditory and proprioceptive information is generally fundamental for mapping external sound and body position, motor command interferes with sound localization. A possible explanation is that active and passive training conditions differ in the exploration of the workspace. Even if the amount of training time was the same, in the passive condition the workspace was entirely explored following two movement modalities (continuous and discrete). On the other hand, in the active training the exploration was free and thus dependent by participants’ movement. Even if participants were required to move along the entire workspace in the active condition, the exploration could have been less consistent compared to the passive training condition. However, in our opinion this difference is not sufficient to explain such finding. An alternative hypothesis is that the motor command does not produce any additional information useful for the sonorous stimulus localization; oppositely, it constitutes a form of “bad noise” which compromises the correct association of auditory and proprioceptive signals. In support of this hypothesis, a study on haptic curvature discrimination demonstrated that a comparison of discrimination curvature between active and passive exploration did not highlight any enhancement in the presence of the motor command (Sciutti et al., 2010). The same experiment conducted on children highlights a negative effect of the presence of motor command on curvature discrimination; haptic precision in children was consistently lower during active exploration when compared to passive motion (Gori et al., 2012b). Consequently, the exploratory movements themselves can constitute a form of noise for the developing haptic system. Thus, motor command can assume the role of noise in auditory perception, negatively affecting the recalibration of the auditory system. However, we did not find any difference between active and passive training on proprioceptive function modification. Both conditions improved proprioceptive accuracy more than 30%. Conversely, a superior effect of the active condition is reported in literature when visual input is provided (Beets et al., 2012). The non-proprioceptive feedback might, in this case, facilitate sensorimotor learning by leading to error detection and movement correction. For instance, when the visual feedback is not provided, as reported in this study, the sensory prediction error is not created, therefore, the final effect on somatosensory function results equal in both active and passive conditions.

### Multimodal vs. Unimodal Training Conditions

The second main finding of this study is that a multimodal training with an auditory feedback congruent with subject’s movement improves spatial perception both in the auditory and the proprioceptive domains, with higher accuracy in reaching a sonorous stimulus and matching arm position. Specifically, we found that relying on solely auditory feedback resulted in a slight, but statistically not significant improvement in sound localization acuity (15%) while relying on audio-proprioceptive feedback increased sound localization acuity by 30%. Similarly, relying on solely proprioceptive feedback results in a slight, but statistically not significant improvement in proprioceptive localization acuity (8.9%) while relying on audio-proprioceptive feedback increased somatosensory acuity by 33.6%. Indeed, we provide evidence that only combined audio-proprioceptive feedback affects both auditory and proprioceptive domains, suggesting that multimodal training based on the congruent association of auditory and proprioceptive feedback can enhance perceptual functions. A possible interpretation of such result is that the multisensory gain could foster the refinement of a coherent audio-motor spatial map that is necessary to orient body in space. Interestingly, we found a mutual influence of the auditory and proprioceptive domains. Indeed, while proprioceptive function benefits from a purely audio training without proprioceptive gain, the purely proprioceptive training mostly affects the auditory domain. This result suggests that external auditory spatial cues can be used to refine the spatial representation of the surrounding environment and implicitly update body movements in space. For instance, we previously demonstrated that even short audio-motor training interventions can improve spatial accuracy in localizing sounds in blind participants due to multisensory enhancement (Finocchietti et al., 2017). The mechanism subtending this multisensory gain could be related to the consolidation of memory processes (Lehmann and Murray, 2005) or the use of selective attention on the trained region (Kaya and Elhilali, 2017). Both these two processes can be involved in the training benefits observed by using a pure audio training.

### Generalization to the Untrained Body Space

The third insight of this study is that the enhancement of spatial localization on the untrained body side is visible only in passive multimodal conditions and only on the auditory domain, indicating that multisensory information helps creating a generalized spatial map that is dependent on memory or attentional processes (Talsma et al., 2010). Indeed it has been previously shown that attention to a sensory modality can affect perceptual estimates in multisensory tasks (Bertelson and Radeau, 1981; Warren et al., 1981; Alsius et al., 2005) and multisensory experiences can improve the encoding and the retrieval of stimuli, e.g., repeated images are better discriminated if initially presented as auditory–visual pairs, rather than only visually (Murray et al., 2004). On the contrary, we found that spatial enhancement following auditory and proprioceptive training (unimodal conditions) did not generalize to the correspondent non-dominant body part, indicating that spatial memory or spatial attention gains are specific for the spatial region trained with the targeted feedback. Similarly it has been showed that proprioceptive acuity improved following motor learning, but only in the region of the arm’s workspace explored during learning, while no proprioceptive improvement was observed when motor learning was performed in a different location (Wong et al., 2011). We can speculate that this short-term sensorimotor plasticity, during which parallel changes to motor and sensory areas occur throughout motor learning (Ostry et al., 2010), is related to the brain hemisphere mainly involved during training. However, we cannot exclude that the transfer to the contralateral part would be visible with a longer or more intensive training session. Nevertheless, it is certain that if the proprioceptive improvement is localized to the trained side, the additional feedback can be provided also on the homologous untrained part, promoting the transferability of feedback information between the two brain hemispheres (Cuppone et al., 2016). Moreover, we cannot exclude that the lack of generalization to the untrained part is due to the constraints of the experimental task. Indeed several reports indicate that inter-limb transfer generally occurs in extrinsic, Cartesian-like coordinates that are not directly from body coordinates (Dizio and Lackner, 1995; Criscimagna-Hemminger et al., 2003). Nonetheless, since the setup orientation was switched from the trained to the untrained side of the body, all the subjects enrolled in our study performed the task in the untrained side according to intrinsic coordinates, thus yielding a mirror transformation of stimuli properties between arms. We can speculate that by maintaining the experimental setup in its original orientation when testing the untrained side, generalization effects might be greater.

## Conclusion

This study evaluated the impact of different sensorimotor training on the improvement of sound localization and body perception in healthy individuals. The results showed that passive guided training with audio-proprioceptive feedback was beneficial to spatial mapping of both auditory and proprioceptive domains. The presence of combined audio-proprioceptive feedback directly affects both auditory and proprioceptive domains: multimodal training based on the congruent association of auditory and proprioceptive feedback can enhance perceptual functions. Interestingly we also observed a strong cross-modal influence of the auditory and proprioceptive domains in training with single feedback (auditory or proprioceptive). These findings highlight the important role of the combined audio-proprioceptive information for auditory and proprioceptive domains, crucial for the execution of daily life activities. Future research should look at the clinical application particularly on subjects with proprioceptive deficits as reported by several neurological diseases (Carey et al., 1993; Rickards and Cody, 1997; Putzki et al., 2006; Konczak et al., 2009; Dukelow et al., 2010) or with deficits in auditory spatial perception as in blindness (Cappagli et al., 2017a). Moreover, investigation of crossmodal correspondences between sensory modalities such as audition and proprioception is of particular interest for the development of pedagogical tools used to informally convey key concepts at school. For instance, our results indicate that sensory modalities other than vision can provide important feedback for the development of spatial perception, which is known to be a prerequisite for learning geometrical concepts.

## Ethics Statement

The study was approved by the ethics committee of the local health service (ASL3 3 Genovese) and parental or adult informed written consent for the study was obtained in all cases before testing subjects.

## Author Contributions

AVC collected, analyzed and interpreted the data and wrote the article. GC helped with the planning of experiments, collected and interpreted the data and wrote the article. MG coordinated the study and the development of the ABBI device.

## Conflict of Interest Statement

The authors declare that the research was conducted in the absence of any commercial or financial relationships that could be construed as a potential conflict of interest.
